# Gender-specific cephalometric features related to obesity in sleep apnea patients: trilogy of soft palate-mandible-hyoid bone

**DOI:** 10.1186/s40902-019-0242-0

**Published:** 2019-12-11

**Authors:** Seok Hyun Cho, Jae-Yun Jeon, Kun-Soo Jang, Sang Yoon Kim, Kyung Rae Kim, Seungho Ryu, Kyung-Gyun Hwang

**Affiliations:** 1 Department of Otorhinolaryngology-Head and Neck Surgery, School of Medicine, Hanyang University, Seoul, Korea; 2 Department of Dentistry/Oral and Maxillofacial Surgery, School of Medicine, Hanyang University, 222-1 Wangshimniro, Seongdong-gu, Seoul, 133-792 Korea; 3 Department of Oral and Maxillofacial Surgery, Massachusetts General Hospital, Harvard Medical School, Boston, USA; 4 Vienna, USA; 5 Department of Occupational Medicine, Kangbuk Samsung Hospital, School of Medicine, Sungkyunkwan University, Seoul, Korea

**Keywords:** Central obesity, Cephalometry, Gender, Sleep disorders, Airway

## Abstract

**Background:**

The aim of this study is to investigate the relationship between gender-specific and obesity-related airway anatomy in patients with obstructive sleep apnea (OSA) by using cephalometric analyses.

**Methods:**

We retrospectively evaluated 206 patients with suspected OSA undergoing polysomnography and anthropometric measurements such as body mass index, neck circumference, and waist-hip ratio. We checked lateral cephalometry to measure tissue landmarks including angle from A point to nasion to B point (ANB), soft palate length (SPL), soft palate thickness (SPT), retropalatal space (RPS), retrolingual space (RLS), and mandibular plane to hyoid (MPH).

**Results:**

Male with OSA showed significantly increased SPL (*P* = .006) compared with controls. SPL and MPH had significant correlation with apnea-hypopnea index (AHI) and central obesity. Female with OSA showed significantly increased ANB (*P* = .013) and SPT (*P* = .004) compared with controls. The receiver operating characteristic curves revealed that SPT in male and ANB and SPT in female were significant in model 1 (AHI ≥ 5) and model 2 (AHI ≥ 15). MPH was also significant for male in model 2.

**Conclusion:**

Male and female with OSA had distinct anatomic features of the upper airway and different interactions among soft palate, mandible, and hyoid bone.

## Background

Obstructive sleep apnea (OSA) refers to sleep-disordered breathing showing repetitive episodes of upper airway collapse during sleep [1]. The risk factors for OSA have been reported as old age, male, smoker, alcohol use, obesity, and craniofacial abnormality [2–6]. Therefore, the pathogenesis of OSA may be related to altered anatomy (small box) and physiology (increased collapsibility) of the upper airway. However, the exact mechanism of OSA has not been fully elucidated. Our previous study demonstrated that a predictor variable neck circumference (NC) is reliable anthropometry for male with OSA [7]. However, it is not clear how this localized obesity in the neck alters the anatomy of the upper airway.

Polysomnography (PSG) is the gold standard test to diagnose the presence of OSA and to estimate its severity. And, apnea-hypopnea index (AHI) criteria with the mean and lowest SpO_2_ are used for treatment planning. However, they do not provide the anatomic locations of obstruction.

Most sleep surgeons perform upper airway imaging studies such as awake and sleep endoscopy, lateral cephalometry, fluoroscopy, computed tomography (CT), or magnetic resonance imaging (MRI) in order to determine surgical versus medical (CPAP, weight control, and oral appliances) management for OSA. The lateral cephalometry is a traditional dental radiograph that shows two-dimensional structures of bony and soft tissues. Riley et al. [8] reported anatomical characteristics of OSA patients with cephalometric analysis in 1983, and thereafter, a series of analyses on OSA patients have been performed to validate its sensitivity as a diagnostic tool for OSA [9–12]. However, the diagnostic values of lateral cephalometry in previous studies remained uncertain. Our hypothesis was that compared with females, males with OSA may have different characteristics of the upper airway which can be influenced by genetics (sex) and environments (central obesity).

This study measured the anthropometry (body mass index (BMI), neck circumference (NC), waist circumference (WC), and waist-hip ratio (WHR)) and obtained a full-night PSG and lateral cephalometry. The aim of this study was to evaluate the relationships between the upper airway anatomy (lateral cephalometry) and anthropometry in patients with OSA. In addition, we investigated the gender-specific and obesity-related anatomy of the upper airway in OSA patients to aid the treatment approach and management.

## Methods

### Patients

There were 288 patients with OSA symptoms (e.g., excessive daytime sleepiness, loud snoring, or observed apnea episodes) who visited the sleep clinic at University Hospital. Exclusion criteria were as follows: (1) patient younger than 18 years (*n* = 5); (2) non-Asian patients (*n* = 10); (3) patients who refused to undergo PSG (*n* = 22); and lateral cephalometry (*n* = 45). A total of 206 patients were included in this study. The symptom questionnaire was used to assess daytime sleepiness (Epworth Sleepiness Scales (ESS)) and sleep quality (Pittsburgh Sleep Quality Index (PSQI)). Detailed medical history was obtained, and anthropometry (BMI, NC, WC, and WHR) was measured prior to PSG. All study protocol was approved by the local Institutional Review Board, and all subjects provided written informed consent (HY-2019-01-007-001).

### Polysomnography

In-laboratory PSG was performed per standard clinical guideline s[13] and included full EEG, EOG, chin EMG, leg EMG, ECG, airflow recorded with nasal/oral thermistor and nasal/oral cannula, pulse oximetry, and body position. According to the American Academy of Sleep Medicine (AASM) Sleep Apnea Definitions Task Force defined in 2012, apnea was scored when there is a drop in the peak signal excursion by ≥ 90% of pre-event baseline using an oronasal thermal sensor lasting ≥ 10 s. Hypopneas were scored when (1) the peak signal excursions drop by ≥ 30% of pre-event baseline using nasal pressure lasting ≥ 10 s and (2) there is ≥ 3% oxygen desaturation from pre-event baseline or the event is associated with an arousal. The AHI was calculated as the number of apnea plus hypopnea per hour of sleep [14]. The cutoff point for OSA was an AHI ≥ 5.

### Cephalometric measurements

**Fig. 1:**
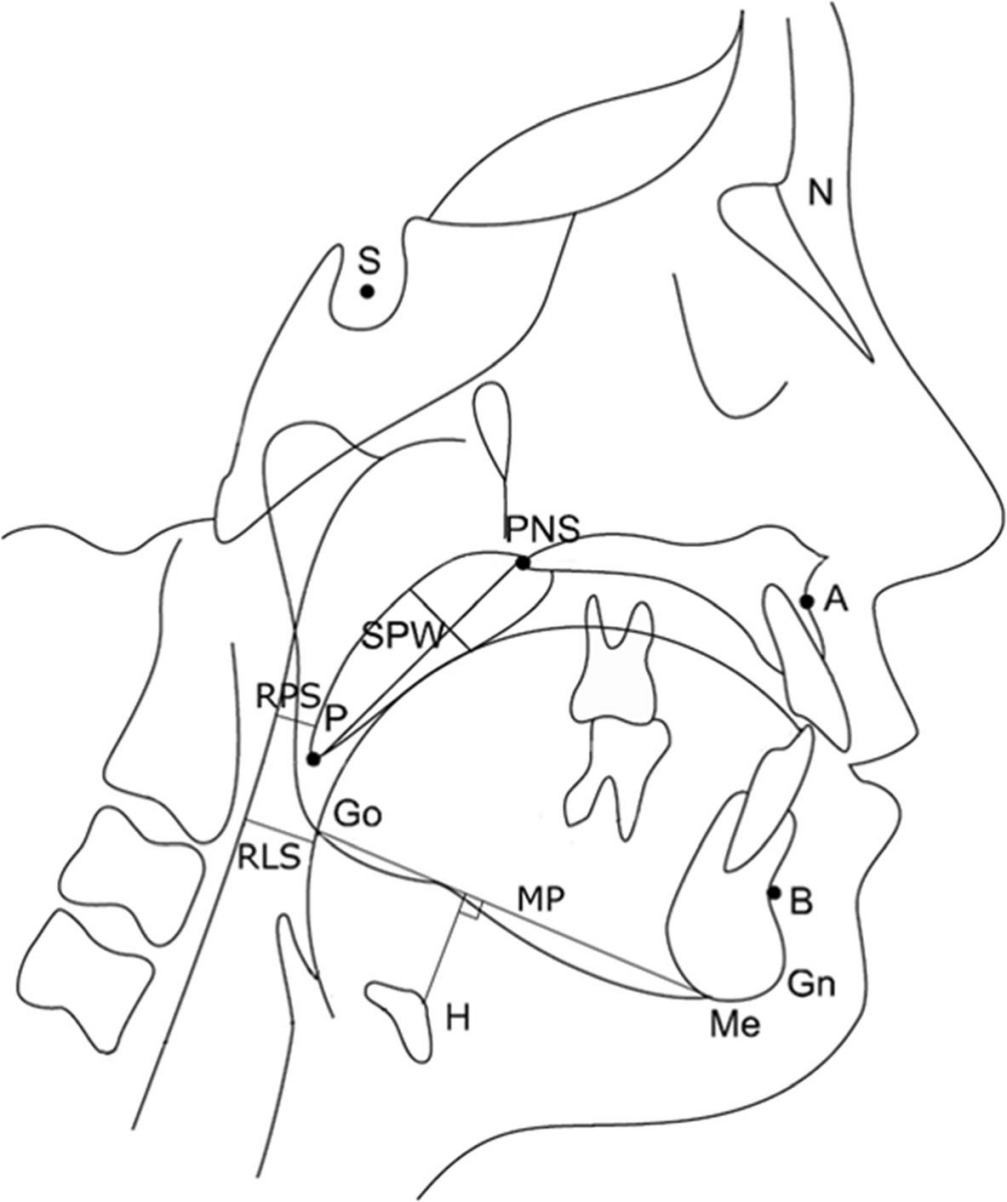
Linear and angular measurements for bony and soft tissue framework in lateral cephalometry. S (sella), midpoint of the fossa hypophysealis; N (nasion), anterior point at the frontonasal suture; A, the deepest anterior point in the concavity of the anterior maxilla; B, the deepest anterior point in the concavity of the anterior mandible; PNS (posterior nasal spine), the most posterior point on the nasal spine; Go (gonion), a mid-point at the gonial angle located by bisecting the posterior and inferior borders of the mandible; Gn (gnathion), the most anteroinferior point on the chin bone; Me (menton), the most inferior point on the chin bone; P, the inferior tip of the soft palate; H, the most anterosuperior point on the hyoid bone; MP (mandibular plane), a tangent line from Me to the inferior mandibular border

Lateral cephalometry was taken after performing PSG and was taken using a standard protocol. All patients were positioned such that the FH plane was parallel to ground level with upper and lower lips, tongue relaxed, and teeth in centric occlusion while holding the breath at the end of the expiratory phase. The length and angular parameters of bony and soft tissue landmarks were measured using the picture archiving and communication system (PACS, Piview STAR, INFINITT, Korea) and analyzed the data using the V-ceph 5.0 software (Cybermed Inc., Seoul, Korea). Cephalometric analyses were corrected for cranio-cervical inclination in order to standardize the data and correction of head position [15]. SNA (angle from sella (S) to nasion (N) to A point), SNB (angle from S to N to B point), and ANB (angle from A point to N to B point) were measured to obtain protrusion angles of maxilla and mandible from the cranium. Posterior airway space (PAS) was measured at two locations: retropalatal space (RPS) and retrolingual space (RLS). Anatomy of the soft palate was measured with its length (SPL) and thickness (SPT). The linear distance along the perpendicular plane from the hyoid bone mandibular plane (MPH) was measured (Table 1 and Fig. 1).

**Table 1:** Cephalometric measurements

Variable	Definition
SNA	Angle from sella to nasion to A point
SNB	Angle from sella to nasion to B point
ANB	Angle from A point to nasion to B point
SPL (soft palate length)	Distance from PNS to tip of the soft palate
SPW (soft palate width)	Widest width along perpendicular line to PNS to tip of soft palate
RPS (retropalatal space)	Shortest distance from soft palate to posterior pharyngeal wall
RLS (retrolingual space)	Shortest distance from tongue base to posterior pharyngeal wall
MPH	Linear distance along the perpendicular plane from Hyoid to mandibular plane

### Statistical analysis

All measured values were presented as mean (standard deviation) for continuous variables and as frequencies and percentages for categorical variables. Student’s *t* test was used to assess the differences among continuous variables, and the categorical variables between genders were analyzed by chi-square test. Pearson’s correlation coefficient was used to determine the correlations between AHI and anthropometric and cephalometric data. Cutoff values and its sensitivity, specificity, and positive and negative predictive values were calculated for each gender. All cephalometric parameters were compared in terms of their receiver operating characteristic (ROC) curves. This method compares the diagnostic properties of the test by expressing sensitivity as a function of 1 – specificity. Results were considered significant at a value of *P* < .05 and with a confidence interval (CI) of 95%. All analyses were performed with Statistical Package for the Social Sciences (SPSS) version 18.0 (SPSS Inc., Chicago, IL, USA).

## Results

Two hundred six subjects were enrolled in the study and Table 2 shows their clinical characteristics with anthropometry, cephalometry, and polysomnography results. OSA groups were older than corresponding control groups without any differences in ESS and PSQI. In male with OSA, BMI, NC, and WHR were significantly elevated than controls and showed significant correlations with AHI (Table 3). However, WHR was the only parameter with higher values in female with OSA and also had a significant correlation with AHI. All patients with OSA showed significantly worsen AHI and lowest nadir SpO_2_ than controls.

**Table 2:** Gender effects on cephalometric differences between OSA and control patients

	Male (*n* = 162)		*P* value	Female (*n* = 44)		*P* value
	OSA	Control		OSA	Control	
	(*n* = 135)	(*n* = 27)		(*n* = 23)	(*n* = 21)	
Clinical						
Age, years	46.4 (13.2)	35.9 (11.1)	0.001	56.0 (12.2)	44.0 (12.1)	0.002
ESS	8.8 (5.2)	9.3 (5.3)	0.613	9.0 (4.9)	9.8 (6.3)	0.655
PSQI	7.3 (3.5)	6.8 (3.3)	0.526	8.5 (4.4)	9.6 (4.3)	0.412
Anthropometry						
BMI, kg/m^2^	25.9 (3.3)	24.6 (3.5)	0.045	25.5 (4.8)	23.4 (3.2)	0.105
NC, cm	39.9 (2.7)	37.9 (4.1)	0.002	35.5 (3.3)	35.1 (6.2)	0.790
WHR	0.95 (0.05)	0.89 (0.05)	0.001	0.91 (0.04)	0.86 (0.05)	0.002
Cephalometry						
SNA	83.9 (5.0)	85.4 (4.6)	0.155	84.3 (5.6)	82.5 (4.8)	0.260
SNB	81.0 (4.8)	82.7 (4.9)	0.099	79.9 (4.9)	80.2 (5.1)	0.850
ANB	2.9 (2.8)	2.7 (2.8)	0.777	4.5 (2.5)	2.4 (2.8)	0.013
SPL	42.4 (4.4)	39.9 (3.9)	0.006	39.9 (5.7)	37.9 (4.5)	0.186
SPT	11.7 (1.9)	11.4 (1.9)	0.553	11.2 (2.1)	9.7 (1.2)	0.004
RPS	7.5 (2.5)	8.5 (2.9)	0.06	6.8 (2.6)	7.1 (2.1)	0.667
RLS	11.0 (3.8)	11.3 (4.2)	0.758	8.9 (2.8)	9.1 (2.5)	0.825
MPH	19.1 (6.4)	18.0 (7.2)	0.453	11.9 (7.1)	12.5 (6.8)	0.777
Polysomnography						
N3, %	1.5 (3.8)	6.0 (9.3)	0.001	3.6 (6.9)	4.7 (5.9)	0.592
AHI, events/h	36.5 (26.3)	2.2 (1.4)	0.001	30.1 (22.0)	1.6 (1.5)	0.001
Lowest O_2_, %	79.9 (10.5)	91.6 (4.1)	0.001	81.2 (8.1)	92.1 (3.2)	0.001

All values are reported as mean (SD). OSA, obstructive sleep apnea; *ESS* Epworth Sleepiness Scale, *PSQI* Pittsburgh Sleep Quality Index, *NC* neck circumference, *WHR* waist-hip ratio, *SE* sleep efficiency, *N3* deep sleep, *AHI* apnea-hypopnea index

**Table 3:** Correlation of anthropometric and cephalometric parameters with AHI

Parameters	All subjects	Male	Female
Anthropometry			
BMI	0.356**	0.368**	0.240
NC	0.361**	0.387**	0.103
WHR	0.428**	0.384**	0.397**
Cephalometry			
ANB	0.079	0.042	0.384*
SPL	0.284**	0.267**	0.141
SPT	0.234**	0.150	0.386**
RPS	− 0.048	− 0.053	− 0.208
RLS	0.137*	0.108	− 0.024
MPH	0.339**	0.292**	0.253

*P < 0.05; **P < 0.01. Numbers are Pearson’s correlation coefficient (*r* values). *AHI* apnea-hypopnea index, *BMI* body mass index, *NC* neck circumference, *WHR* waist-hip ratio, *ANB* angle from A point to nasion to B point, *SPL* soft palate length, *SPT* soft palate thickness, *RPS* retropalatal space, *RLS* retrolingual space, *MPH* mandibular plane to hyoid

### Gender-specific characteristics of the upper airway anatomy in OSA

Male and female with OSA had different cephalometric profiles (Table 2). In male, SPL (*P* = .006) was significantly increased in the OSA group but there was no difference in other parameters. However, in female, ANB (*P* = .013) and SPT (*P* = .004) were significantly higher in the OSA group but there was no difference in other parameters.

### Gender differences in associations between the upper airway anatomy and AHI

Male and female showed different associations between the upper airway anatomy (lateral cephalometry) and AHI (Table 3). In male, SPL (*r* = .267, *P* = .001) and MPH (*r* = .292, *P* = .001) showed significant correlations with AHI. However, in female, SPT (*r* = .386, *P* = .01) showed a significant correlation with AHI.

### Gender-specific effects of central obesity on the upper airway anatomy

Central obesity had different impacts on the upper airway anatomy measured with lateral cephalometry (Table 4). In male, NC showed significant correlations with SPL (*r* = .208, *P* = .008), SPT (*r* = .3, *P* = .001), RLS (*r* = .168, *P* = .033), and MPH (*r* = .161, *P* = .042). And WHR showed significant correlation with SPT (*r* = .211, *P* = .007) and MPH (*r* = .162, *P* = .04). However, in female, none of the cephalometric indexes had any correlations with NC or WHR.

**Table 4:** Effect of central obesity (anthropometry) on the upper airway anatomy (cephalometry)

Parameters	All subjects	Male	Female
Correlation with NC			
ANB	− 0.120	− 0.124	0.014
SPL	0.197**	0.208**	− 0.151
SPT	0.305**	0.300**	0.075
RPS	0.022	− 0.028	− 0.037
RLS	0.168*	0.168*	− 0.194
MPH	0.314**	0.161*	0.231
Correlation with WHR			
ANB	− 0.046	− 0.066	0.210
SPL	0.177*	0.126	− 0.033
SPT	0.258**	0.211**	0.104
RPS	0.067	0.047	− 0.048
RLS	0.186**	0.136	0.025
MPH	0.245**	0.162*	0.002

*P < 0.05; **P < 0.01. Numbers are Pearson’s correlation coefficient (*r* values). *NC* neck circumference, *WHR* waist-hip ratio, *ANB* angle from A point to nasion to B point, *SPL* soft palate length, *SPT* soft palate thickness, *RPS* retropalatal space, *RLS* retrolingual space, *MPH* mandibular plane to hyoid

### Cutoff values and areas under the ROC curve of lateral cephalometry in male and female with OSA

**Fig. 2:**
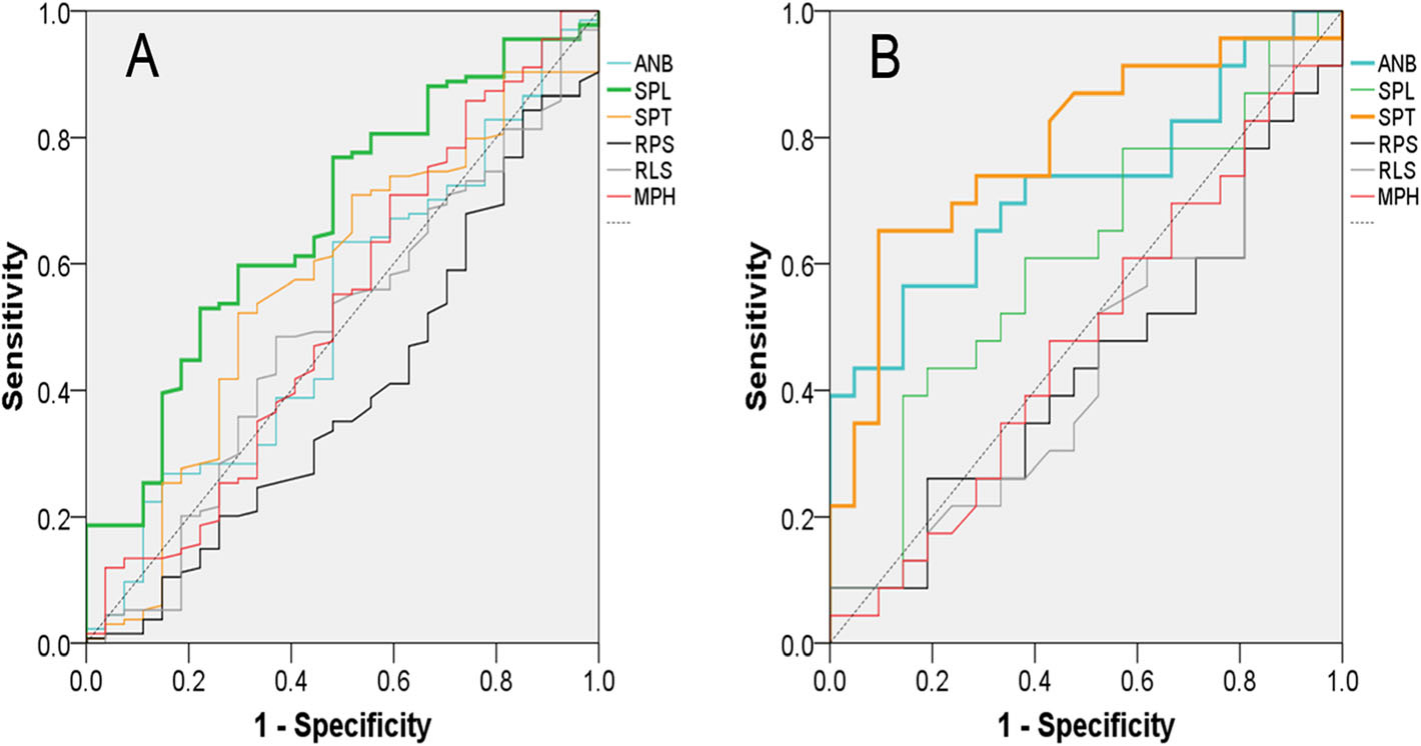
Receiver operating characteristic curves in male (**a**) and female (**b**) for angle from sella to nasion to A point (ANB), soft palate length (SPL), soft palate thickness (SPT), retropalatal space (RPS), retrolingual space (RLS), linear distance along the perpendicular plane from hyoid to mandibular plane (MPH)

In male and female, ROC curves were constructed for each cephalometric factor to evaluate their diagnostic power to OSA and calculated cutoff values (Fig. 2). Table 5 gives the areas under the ROC curve for cephalometric variables and their significance in predicting OSA. In model 1 (AHI ≥ 5), the areas under the ROC curve for male with OSA were significant for SPL (*P* = .005). In female, the areas under the ROC curve for OSA were significant for SPT (*P* = .001). In model 2 (AHI ≥ 15), both SPL (*P* = .004) and MPH (*P* = .001) were significant for male with OSA. However, in female, SPT (*P* = .003) remained significant for OSA.

**Table 5:** Comparison of areas under the receiver operating characteristic curves in male and female

	Male		Female	
	AUC	*P* value	AUC	*P* value
Model 1 (AHI ≥ 5)				
ANB	0.519	0.751	0.725	0.011*
SPL	0.670	0.005**	0.605	0.235
SPT	0.568	0.265	0.784	0.001**
RPS	0.396	0.087	0.437	0.474
RLS	0.493	0.912	0.452	0.589
MPH	0.527	0.656	0.477	0.796
Model 2 (AHI ≥ 15)				
ANB	0.547	0.310	0.736	0.009**
SPL	0.635	0.004**	0.498	0.981
SPT	0.568	0.144	0.772	0.003**
RPS	0.481	0.675	0.461	0.090
RLS	0.549	0.294	0.511	0.089
MPH	0.648	0.001**	0.523	0.094

*P < 0.05; **P < 0.01. *AUC* area under the ROC curve, *AHI* apnea-hypopnea index, *ANB* angle from A point to nasion to B point, *SPL* soft palate length, *SPT* soft palate thickness, *RPS* retropalatal space, *RLS* retrolingual space, *MPH* mandibular plane to hyoid

## Discussion

This study investigated the anatomic variants of the upper airway (lateral cephalometry) related to sex and central obesity in Asian patients with OSA. The different phenotypes of OSA determined by sex and central obesity may permit us to make appropriate therapeutic plans for OSA patients. The key findings of this study were as follows: (1) Soft palate was found to be the main target of OSA and its anatomic changes show significant correlations with AHI. (2) Phenotypes of soft palate were different between sexes: increased length for male and increased thickness for female. (3) Only male OSA patients showed clear associations between central obesity and the upper airway anatomy. (4) MPH was a male-specific parameter especially for moderate and severe OSA and showed a significant correlation with both AHI and central obesity.

Male with OSA showed typical characteristics of central obesity with increased NC and WHR. There have been many studies demonstrating the altered neck anatomy in male with OSA including increased neck circumference (anthropometry) and parapharyngeal fat deposition (CT or MRI) [16–18]. Therefore, it is reasonable to state that central obesity (thick neck) in male with OSA may cause the altered anatomy of the upper airway. To illustrate OSA-related upper airway anatomy, a total of eight well-known parameters on lateral cephalometry were used for craniofacial anatomy (SNA, SNB, and ANB), upper airway (SPL, SPT, and RPS), and lower airway (RLS and MPH). Lateral cephalometry does not show that the volume of airway and cephalometric analysis was affected by the head position. Nevertheless, it has been used in maxillofacial deformity analysis and orthodontic diagnosis. The lateral cephalometry has been used for its simplicity and economical method for measuring anatomical structure related to the airway. In this study, we standardized the data by correcting the cranio-cervical inclination in order to obtain accurate measurements of parameters of the airway. This study clearly demonstrated that the upper airway anatomy measured with lateral cephalometry had different characteristics and associations with AHI and central obesity, depending on sex.

SNA, SNB, and ANB were used to evaluate the anterior-posterior position of maxilla and mandible to cranial base on lateral cephalometry. Retrognathia is well-known anatomic phenotype affecting the pathogenesis of OSA [19–22]. Lowe et al. [21] showed that patients with OSA had the smaller and posteriorly positioned mandible which decreased the overall airway space on lateral cephalometry. In contrast, others reported that there was no difference in upper airway volume based on the intermaxillary relationship using cone beam computed tomography (CBCT) [23]. However, our study demonstrated that mandible was positioned posteriorly in female with OSA (increased ANB) which was independent with obesity and had significant correlations with AHI. Thus, retrognathia is an important anatomic phenotype affecting the presence and severity of OSA in female.

**Fig. 3:**
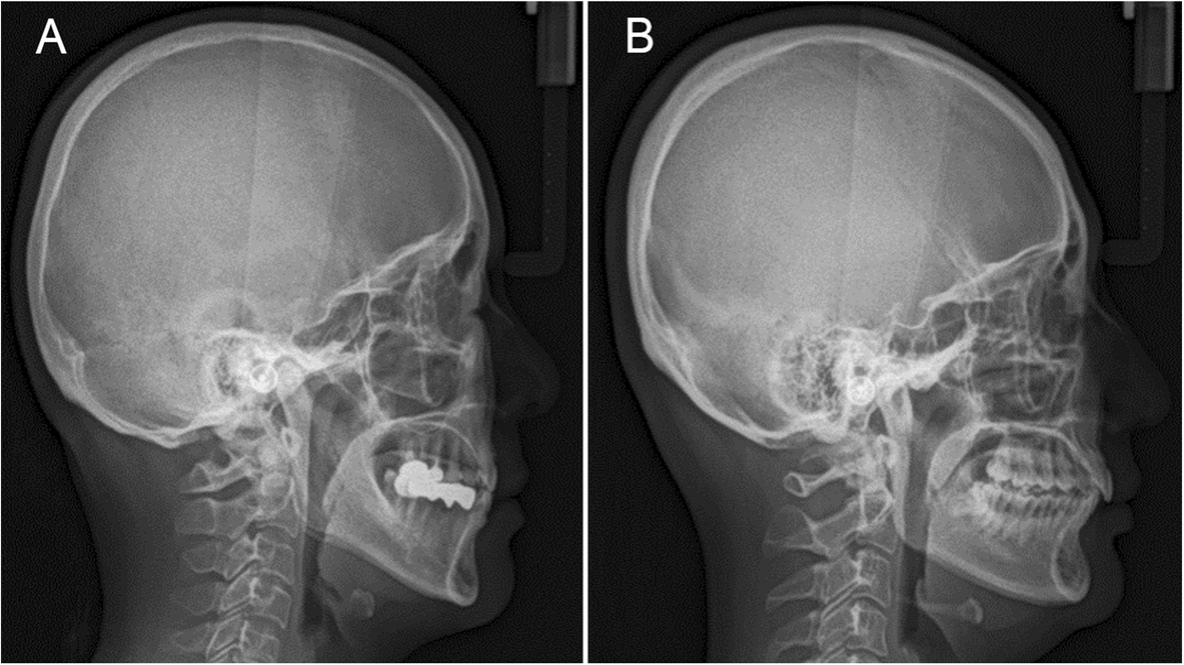
Representative views of lateral cephalometry show the different morphology of the soft palate, mandible, and hyoid bone between male (**a**) and female (**b**) with obstructive sleep apnea

There are many reports using lateral cephalometry to investigate the influence of soft tissues on OSA. Previous studies reported that increased soft palate length and thickness are associated with OSA on lateral cephalometry [21, 24–27]. In the study targeting 62 males, Yu et al. [28] also reported that longer SPL was associated with higher prevalence of OSA. In contrast, Cillo et al. [29] reported that there were no significant changes in SPL in OSA. However, in this study, we found that male with OSA had a different phenotype of the soft palate compared with female with OSA. Male with OSA had significantly increased SPL which showed significant association with AHI and NC. Alternatively, female with OSA had significantly increased SPT which showed significant association with AHI but not with central obesity (NC and WHR). Therefore, the soft palate may become elongated in conjunction with the weight gain which can aggravate the upper airway narrowing in male. However, female may have an intrinsic variance of SPT which is independent with central obesity. Female with thick palate may have increased risk for the development of OSA (Fig. 3).

Retro-palatal and retro-lingual spaces are the most common sites of upper airway narrowing in OSA [30, 31]. Dynamic studies such as drug-induced sleep endoscopy (DISE) also reported that there are isolated or multiple sites of upper airway collapse in OSA [32]. However, in our cephalometric study, there was no difference in RPS and RLS between OSA and controls regardless of the sex. Moreover, they did not show any correlation with AHI and central obesity. Thus, we concluded that 2-dimensional analysis of the upper airway space may have limitations to represent OSA. To investigate the space anatomy, upper airway endoscopy may be advantageous to show 3-dimensional views and dynamics in OSA.

The position of the hyoid bone is related to the retro-lingual space, and lower hyoid indicates the narrowing of RLS and also is associated with weak tonicity of the genioglossus muscle. De berry et al. [33] reported that the hyoid bone in the lower position could displace the base of the tongue further downward leading to the airway obstruction of hypopharynx more easily. However, in our study, there was no difference in MPH between OSA and controls regardless of sex. By subgroup analysis, MPH in male showed significant associations with AHI and central obesity (NC and WHR). Moreover, ROC curves showed that MPH is an anatomic factor representing the moderate-to-severe OSA in male. Whittle et al. [16] reported that male had larger soft tissue volume around the airway compared with female which is consistent with our study. Thus, both intrinsic soft tissue volumes of the upper airway and obesity-related central fat deposition (NC) may affect the MPH in male. By comparison, MPH in female did not show any relationship with AHI and obesity, and therefore, the narrowing of RLS may be not involved in the pathogenesis of female OSA.

The authors acknowledge the weaknesses and limitations of our study. The lateral cephalometry was taken in a standing position and awake state which does not represent accurate measurements of the upper airway in the sleeping state. Also, the number of female was relatively fewer than male. Despite the above-mentioned limitations, our study clearly demonstrated the role of gender on the upper airway anatomic variation and its association with central obesity.

## Conclusion

Phenotypes of the upper airway in OSA differ with the sex. Increased SPL and MPH contributed to OSA in male. However, retrognathia and increased SPT are involved in the pathogenesis of OSA in female. Altered upper airway anatomy is significantly associated with central obesity in male, but these interactions were not found in female.

Male and female may have distinct upper airway anatomy, and different risk factors are involved in the pathogenesis of OSA. Central obesity may increase SPL and MPH and, therefore, aggravate the upper and lower airway collapse in OSA. By comparison, developmental retrognathia and thick soft palate independent with obesity may affect OSA in female.

Our study demonstrated the hidden and useful features of lateral cephalometry for the evaluation of the upper airway in OSA. When considering sex-specific therapeutic plan for OSA, obesity-susceptible upper airway narrowing may indicate the effectiveness of weight control for male with OSA. And retrognathia and thick palate in female may predict the lack of success rate of the palate surgery without tonsillar hypertrophy. Further study will be necessary to demonstrate the values of these anatomy-based treatment plans for OSA.

## Data Availability

Data sharing is not applicable to this article as no data sets were generated or analyzed during the current study.

## Abbreviations

ANB: A point to nasion to B point
BMI: Body mass index
CPAP: Continuous positive airway pressure
MPH: Mandibular plane to hyoid
OSA: Obstructive sleep apnea
PSG: Polysomnography
RLS: Retrolingual space
RPS: Retropalatal space
SPL: Soft palate length
SPT: Soft palate thickness
